# Does Playing Video Games Increase Emotional Creativity?

**DOI:** 10.3390/ijerph17072177

**Published:** 2020-03-25

**Authors:** Inna Čábelková, Wadim Strielkowski, Anna Rybakova, Alla Molchanovа

**Affiliations:** 1 Department of Trade and Finance, Faculty of Economics and Management, Czech University of Life Sciences Prague, Kamýcká 129, 16500 Prague, Czech Republic; 2 Department of Social, General and Clinical Psychology, Russian State Social University, 4/1 Wilhelm Pieck str., 129226 Moscow, Russian Federation; anya_rybakowa@list.ru; 3 Department of Psychology and Pedagogy of Education, Moscow Psychologic-Social University, 9a Roshchinsky 4th Passage, 115191 Moscow, Russian Federation; alla_molchanova67@mail.ru

**Keywords:** emotional creativity, video games, public health, digital technology, gender

## Abstract

Emotional creativity (EC), which constitutes a main aspect of the general creativity concept, is often shown to be substantially related to positive emotional experiences, effective motivation, and innovation at the workplace as well as at school. However, little is known about the relationship between emotional creativity and the time people tend to spend playing video games. Nowadays, video games and virtual reality have become an important aspect of public health and psychological research. They constitute a key element of popular culture and generate considerable economic profit as part of the public entertainment industry. Our study is based on the results of an emotional creativity inventory (ECI) survey that was administered at a snowball and convenient sample of 453 respondents (aged 18–60 years, M ± SD: 23.68 ± 6.36; 66.40% women, 20.00% with higher education) from the Czech Republic who were questioned about their habits and the time they devote to playing video games. The sample country was selected as one with a long tradition of homebrew video gaming going back to the 1980s. We employed a set of multinomial regression analyses, which revealed that more time devoted to playing video games is associated with lower emotional creativity, in general, as well as with lower novelty, preparedness, and effectiveness components of emotional creativity. The negative association above was less pronounced for women than for men. Moreover, in a sample containing only women, a longer time devoted to playing video games was associated with the higher novelty component of emotional creativity (the associations with the other two components were not statistically significant for women only). Our findings might considerably contribute to the study of the general overall long-term effect of video games and the use of digital technologies in general on public health.

## 1. Introduction

Creativity can be seen as one of the main traits of human capacity that is indispensable for the development of knowledge society and innovations [1,2,3]. Emotional creativity (EC) represents one of three main areas of general creativity, which reflects originality and appropriateness in emotional experience [4]. According to Averill [5], three components of EC can be distinguished: (i) Preparedness (understanding and learning from one’s own and other’s emotions), (ii) novelty (ability to experience unusual emotions), and (iii) effectiveness authenticity (expressing emotions honestly, leading to the benefits of the individual or the group).

As part of emotion regulation, EC also contributes to relationship satisfaction [6,7], the overall state of well-being [8,9], and life satisfaction [10]. EC per se has been found to be associated to positive emotional experiences [11,12], including happiness, and life satisfaction [13]. EC also supports behavioral self-regulation, as Fuchs et al. point out [14], and is related to a non-confrontational, democratic, and independent personality [15,16].

EC is often shown to have a significant effect on the innovative performance of employees in a business as, for example, Wang et al. demonstrated [17], and is a significant predictor of intrinsic motivation and engagement. In the educational setting, it was shown to be positively related to self-efficacy and academic motivation [18], educational adjustment [19], and communicative competences. It is also positively related to coping strategies [20] and to the ability to moderate the relationship between supervisors’ paternalistic leadership and teachers’ teaching efficacy in the process of teaching English, which Song described in a case study from China [21].

Educating for creativity in schools and supporting creativity in other ways is felt as one of the major priorities in educational strategies [22,23]. In the workplace, employee creativity is considered to be a key driver of innovation and organizational success and many efforts have been made to study the factors that support workplace creativity [24,25]. In this paper, we argue and provide analytical evidence from a very comprehensive and thorough data set collected in the Czech Republic (which has a history of gaming dating back to the socialist Czechoslovakia as a representative of EU video gaming culture) that playing video games might be one of the factors lowering emotional creativity. Although our results might be limited due to the sample selection, the geographical location, and the methodology (see Section 5.1 explaining all of these in detail), some of our findings might be useful for practitioners, stakeholders, and researchers alike.

Nowadays, in our digital and interconnected world, playing video games has become part of everyday life for people worldwide from an early age [26]. Up to three-quarters of 6- to 15-year-old individuals in Germany, Spain, Italy, the United Kingdom, and France are frequent video game players [27]. In the European Union (EU), a population cohort aged 6–64 years old, the numbers of video games players reached 54%, which equates to some 250 million players in the EU, with the average weekly time of play reaching 8.7 h [28,29].

Due to the growing participation of the EU population in video gaming, the scientific community has studied the positive and negative effects, predispositions, and correlates of video games. Playing video games was shown to be positively related to performance in mathematics, engineering and science [30], and visual-spatial skills (see Green and Bavelier [31] for the case study of adults, or Jackson et al. [32] for the case study of children), to increased odds of high intellectual functioning and overall school competence [33]. Several studies confirmed that playing preferred video games leads to an increase in positive emotion [34,35], improves players’ moods, promotes relaxation, and helps to ward off the anxiety [36].

On the other hand, some research showed that playing video games (alongside the use of gadgets, such as smartphones, tablets, and game consoles, to access other media sources, such as the Internet and TV) can be attributed to sadness, depression and an increased risk of suicide [37,38], insensitivity of children to violence [39], and aggressive behavior [40,41]. Moreover, it was shown that intensive use of media sources, playing game included, was also related to increased obesity risks [42].

This paper aimed to study the association between the time the respondents spent playing video games and emotional creativity, including its various components. We tested the relation between EC and its four components on the one side and the time devoted to playing video games on the other on a sample of 453 respondents (aged 18–60 years, M ± SD: 23.68 ± 6.36; 66.40% women, 20.00% with higher education) from the Czech Republic, a country that has complex relationships with more influential centers of game development and gaming communities and has been distinguished by a homebrew computer gaming culture since the Socialist period of the 1980s and then in the early 1990s (see, e.g., an interesting study by Šisler et al. on that topic [43]). Given that the play time of men and women differ and there are statistically significant gender differences in emotional creativity [44], we also studied the moderation effect of gender. The following hypotheses were developed for testing in this paper:

**Hypothesis** **1.** *Emotional creativity is associated with the time devoted to playing video games*.

**Hypothesis** **1a.** *The association above is moderated by gender*.

**Hypothesis** **2.** *All the four components of emotional creativity (novelty, preparedness, effectiveness, and authenticity) are related to the time devoted to playing video games*.

**Hypothesis** **2a.** *The association above is moderated by gender*.

In this paper, we studied how the time devoted to playing video games is associated with emotional creativity as a total (either lowering it or increasing it). Moreover, we tested the impact of novelty and preparedness components of emotional creativity. In addition, we determined whether this association is more pronounced for women or to men. This paper is organized as follows: Section 2 presents the basic information about the participants and survey. Furthermore, it presents the variables and our empirical model. Section 3 outlines the main results. Section 4 provides an in-depth discussion of the results. Finally, Section 5 closes the paper with some conclusions and implications.

## 2. Materials and Methods

### 2.1. Participants and Survey

The data used for testing the empirical model presented in this study come from the Czech Republic. The country has been known as the center of the hobby computer culture and homebrew video games since the 1980s [45]. A total of 460 respondents completed a questionnaire voluntarily and anonymously. All participants were Czech native speakers living in the Czech Republic. The method of sampling included elements of the snowball technique and opportunity sampling. The participants were recruited via e-mail. Due to the methodology of the sample construction described above, our sample does not have an ambition to be representative per se. Seven participants were excluded from further analysis due to incomplete or unreadable answers. The resulting sample included 453 respondents (aged 18–60 years, M ± SD: 23.68 ± 6.36; 66.40% women, 20.00% with higher education).

All questionnaires were filled out via the web page available on the Internet. The reason behind this approach is the ability to reach the Internet-based population, which otherwise would not be reachable [46,47], and enables communication with people who are reluctant to meet face to face [48]. Internet-based research helped balance the sample with respect to the time the respondents play video games. Namely, it helped to increase the participation of the heavy players (10% of respondents play more than 3 h daily) to balance the number of non-players (23.6% of the sample, see Table 1). All participants were informed that the data they provided was confidential and used for research purposes only, and would not be transferred to third parties. All subjects gave their informed consent for inclusion before they participated in the study. The study was conducted in accordance with the Declaration of Helsinki, and the protocol was approved by the Ethics Committee of the Czech University of Life Sciences Prague, project No. VEV0306/2019. All the participants participated voluntarily and anonymously in the present study. All the participants signed the informed consent with participation in the study.

### 2.2. The Time Spent Playing Video Games

The respondents were asked how much time they devoted to playing video games. Namely, they were asked to select one option (see Table 1) that best corresponded to their experience. The normative naming of the groups (frequent players, regular gamers, infrequent players, occasional players, non-gamers) was omitted from the questionnaire. The group division and naming were inspired by a study of Ip et al. [49]. The resulting gamer groups and the numbers of the respondents are listed in the Table 1.

In a further analysis, the time spent playing video games, being an ordinal variable, was transformed by taking a smoothing spline approach (Spline Ordinal, Degree 2, Interior Knots 2) in order to avoid loss of ordinality, which was inevitable if we included this variable as a set of dummies in the further analysis [50]. The variable transformation, as opposed to a set of dummies, also enabled us to include the interaction effect of gender versus time played into the further analysis. The resulting Pearson correlation of the former and transformed variable was 0.972 (Sig. 0.000).

### 2.3. Emotional Creativity

The emotional creativity inventory (ECI) self-report questionnaire was used to measure emotional creativity. It consists of 30 items rated on a 5-point scale, which ranges from 1 (strongly agree) to 5 (strongly disagree). The ECI scores ranged from 48 to 138 and averaged at M ± SD: 99.82 ± 15.50. The internal reliability index (Cronbach’s ɑ) reached 0.85, which denotes very good internal reliability.

The ECI contains three components that reflect different aspects of emotional creativity. The preparedness component (range: 8–35, M ± SD: 25.81 ± 5.65, Cronbach’s ɑ: 0.81) includes 7 items. It represents the capability of understanding and learning from one’s own and other’s emotions. The novelty component (range 20–68, M ± SD: 46.83 ± 8.90, Cronbach’s ɑ: 0.79) comprises 14 items. It covers the ability to experience unusual emotions. The effectiveness/authenticity component (range 10–43, M ± SD: 27.18 ± 6.02, Cronbach’s ɑ: 0.74) contains 9 items and is composed of effectiveness (5 items) and authenticity (4 items). It comprises the skills to express emotions leading to a potential benefit to the individual or group.

We also computed the statistics for the effectiveness and authenticity components separately. The effectiveness component (M ± SD: 11.57 ± 3.27, Cronbach’s ɑ: 0.65) included 5 items and ranged from 4 to 20. The authenticity component (M ± SD: 12.51 ± 3.30, Cronbach’s ɑ: 0.66) included 4 items and ranged from 5 to 20. The lower reliability of the effectiveness and authenticity components taken separately as measured by Cronbach’s ɑ is partly given by the low number of items within the component [51]. Still, both scales exhibited acceptable reliability. As all the components yielded good internal reliability, they were used separately in the later analysis.

### 2.4. Sociodemographic Variables

In addition to emotional creativity and video game play time, the respondents were also asked to reveal their age, gender, and education.

Before conducting the actual analysis, we first computed the descriptive statistics and correlations between all the continuous and ordinal variables as well as reliability statistics for ECI and its components. The results are presented in Table 2.

### 2.5. The Model

To test Hypotheses 1, 1a, 2, and 2a predicting the relationships between ECI and its components on one side and play time for video games, a set of multiple linear regression analyses was employed. ECI and its four components (effectiveness and authenticity were used both as one variable and separately) served as dependent variables subsequently. The independent variables comprised the time devoted to video games, the interaction variable of time and gender, age, gender, and education (see Equation (1)). Given that all the explanatory and control variables can affect the target variable simultaneously, and to correct for the effects of common variance and filter out the possible effects of third variables, all explanatory variables were included in the regressions simultaneously. Since some of the independent variables were significantly correlated and as the interaction variable was included, we also computed the Variance Inflation Factors (VIFs) to test for possible multicollinearity:

*ECI_i_* = *a*_0_ + *a*_1_*PlayTime* + *a*_2_PlayTime*Gender + *a*_3_*Age* + *a*_4_*Gender* + *a*_5_*Education* + *ξ*, (1)

where: *ECIi* denotes the indicators of ECI and its components (preparedness, novelty, effectiveness/authenticity, effectiveness, and authenticity); *PlayTime* stands for the time spent playing video games transformed taking a smoothing spline approach (Spline Ordinal, Degree 2, Interior Knots 2, see section on time spent playing video games); *Education* represents educational dummies; and *Age* and *Gender* are the age and gender of the respondents.

## 3. Results

### 3.1. The Effect of Play Time on Emotional Creativity, and its Novelty and Preparedness Components Moderated by Gender

The results of a set of regressions for ECI, ECIn, and ECIp as dependent variables in the regressions (Equation (1)) are presented in Table 3.

All three regressions presented in Table 3 were statistically significant. The variance inflation factors (VIF) are lower than 10, which suggests no serious multicollinearity problem. From Table 3, the following outcomes can be drawn:

- More time devoted to playing video games is associated with lower emotional creativity as a total, and to lower novelty and preparedness components of emotional creativity in particular (the relevant coefficient is positive, but the play time was coded reversely, with lower numbers meaning more play); and
- The association between play time and ECI, ECIp, and ECIn is less pronounced for women than men. In fact, the values of the relevant interaction coefficients (Play time * Gender) suggest that the relationship for a subsample of women may not exist or be the opposite to that of men.

In order to study it further, the regressions below (Equation (2)) were run for the sample of women only:

*ECI_i_* = *a*_0_ + *a*_1_*PlayTime* + *a*_2_*Age* + *a*_3_*Education* + *ξ*, (2)

where *ECIi* denotes the indicators of ECI and its components (preparedness, novelty, effectiveness/authenticity). This regression was run for the sample of women only.

Out of the four regressions above (considering ECI and its three components as dependent variable gives four regressions), only the ECI novelty regression was statistically significant. Table 4 presents the results of the ECI novelty regression.

According to Table 4, higher play time was associated with a higher novelty component of ECI for the sample of women only.

### 3.2. The Effect of Play Time on Authenticity, Effectiveness, and Joint Authenticity/Effectiveness Components of Emotional Creativity Moderated by Gender

The results of the regression analyses are presented in Table 5.

All three regressions presented above are statistically significant; however, the first two are close to the edge of significance. The variance inflation factors (VIFs) are lower than 10, which suggests no serious multicollinearity problem as pointed out in O’Brien [52]. As it follows from Table 5, the following implications can be drawn:

- More time devoted to video games is related to a lower effectiveness component of ECI;
- The moderating effect of gender did not prove to be statistically significant; and
- Women are statistically less emotionally creative in the effectiveness and authenticity/effectiveness components compared to men.

### 3.3. Emotional Creativity, Age, Gender, and Education

Though it was not the aim of this paper to study the relationship between the indicators of emotional creativity and sociodemographic variables (age, gender, and education), some statistical results are worth mentioning:

- The results depicted in Table 3, Table 4 and Table 5 show that: Older respondents report a lower novelty component of emotional creativity (ECIn);
- Women are more emotionally creative according to the components of novelty (ECIn) and preparedness (ECIp) and less emotionally creative according to effectiveness/authenticity (ECIea) and effectiveness (ECIe) compared to men; and
- Respondents with secondary education are more emotionally creative compared to primary educated respondents according to the ECI total.

## 4. Discussion of Results

Our paper identifies novel insights in the association between the time devoted to playing video games and emotional creativity. Surprisingly, in most of the cases, the association was negative, meaning that more time devoted to video games is associated with lower emotional creativity and some of its components (preparedness, novelty, effectiveness) for the whole sample.

Emotional preparedness involves thinking about one’s own emotional reactions and emotional experiences, searching for the reasons for one’s own feelings, or paying attention to other people’s emotions in an effort to better understand one’s own feelings. Thus, the results indicate that either playing video games diverts people from thinking of their emotions or people who are more prone to devoting time to playing video games are less likely to dive into their emotions or the emotions of others.

Emotional novelty covers the ability to experience new unusual emotions, different from the emotions of other people. Within this context, the results suggest that either the videogame play leads to a flattening and standardization of emotional experiences, or people who produce less new emotions are more likely to engage in video games.

Emotional effectiveness implies the skill to employ emotions in order to achieve one’s goals in life and better relationships with others. Moreover, the results suggest that either playing video games leads to a lower ability to employ one’s emotions for the benefit of oneself or others, or people who lack this capacity are intrinsically attracted to spending time playing video games.

Interestingly, the effects above were moderated by gender. Furthermore, in the sample of women only, playing video games was associated with a higher novelty component of emotional creativity. Here, we might suggest that women, as they are less prone to engage in video games than men, might find video games to support the variability and novelty of their emotional experiences, or, to the contrary, women who are inclined to experience new emotions are more prone to engaging in video games.

Overall, the results are in accordance with the existing research literature that suggests mostly negative long-term emotional effects of playing video games, such as sadness, depression, increased risk of suicide (see, e.g., Messias et al. [37] or Wenzel et al. [38]), insensitivity of children to violence (see, e.g., Funk, [39]), and aggressive behavior (Anderson et al. [40] or Bushman and Anderson [41]), although the immediate effects might be positive (Russoniello et al. [36], Ryan et al. [34]). On the other hand, the effects of video games on cognition and intellectual skills, such as performance in mathematics (see, e.g., Subrahmanyam et al. [30]), engineering and science, visual-spatial skills (see, e.g., Green and Bavelier [31] or Jackson et al. [32]), high intellectual functioning, and overall school competence (see, e.g., Kovess-Masfety et al. [33]), are largely positive.

Even though this study was designed to research the causality of the association between emotional creativity and the time spent playing video games, it showed that more research is needed in this direction. Similarly, there might also be a need for specifying the effects of the particular types of video games on emotional creativity. It might also be interesting to look into the mobile apps and games that are gaining popularity over the games played on PCs and game consoles (the best and most successful and hence widely discussed examples of those might be Pokémon Go or Harry Potter: Wizards Unite).

## 5. Conclusions

Overall, one can see that human creativity and playing video games are two topics that have attracted increased research and public attention at all levels. While creativity itself is considered to be indispensable for new knowledge society and innovations, emotional creativity as a part of overall creativity significantly contributes to building human relationships, achieving life satisfaction, and obtaining an overall state of well-being. At the same time, the phenomenon of playing video games has attracted significant attention from the European population due to the fact that for some individuals, it occupies a considerable part of their time. According to some estimates, the numbers of video game players in the EU reached 54% of the population, which equates to some 250 million, people with almost nine hours of video game playing time per week.

While the effects of video games on intellect and emotions, and the ways to improve people’s creativity as separate subjects have received significant research attention, to our knowledge, the association between emotional creativity and time spent playing video games is not well reflected in the research literature.

Our paper studied the association between the time the respondents spent playing video games and their emotional creativity as a whole and partially as its components, such as preparedness, novelty, effectiveness, and authenticity. Our sample consisted of 453 respondents (aged 18–60 years, M ± SD: 23.68 ± 6.36; 66.40% women, 20.00% with higher education) from the Czech Republic, a country that has a long tradition of video games dating back to the socialist Czechoslovakia of the 1980s. Due to the fact that the play time of men and women differs and there are statistically significant gender differences in emotional creativity, we also studied the moderation effect of gender in playing video games.

Our results suggest that extended time playing video games is associated with lower emotional creativity as a whole, and as novelty and preparedness and effectiveness components, in particular. It also appears that this effect is more pronounced for men than for women. In the case of the novelty component and for women only, the effect of playing video games is positive—the more time women play, the higher the novelty component of their emotional creativity.

We can also state that our results are in accordance with the research literature devoted to the effects of playing video games, which suggests generally negative long-term effects on different sides of emotions, even though the immediate emotional effect might be positive, with some positive effects on cognition and intellect. The result of the positive association between the time invested playing video games and the novelty component of emotional creativity in women goes against the generally negative associations with emotions.

In addition, we have to say some words about the pathways of further research on this interesting topic. While the associations above are revealing and significant, more work needs to be done on the causality of the associations. It is not clear whether playing video games produces lower emotional creativity or whether people with inherently low emotional creativity are more attracted to playing video games. The other avenue for further research may concentrate on the effects of different types of video games. In addition, samples from other countries, both with a profound history of video gaming and without one, might be studied in order to figure out the impacts of cultural aspects.

Our results might be informative for stakeholders dealing with public health and digital culture. Video gaming has become an indispensable part of many people’s lives whether we like it or not, hence the effects of gaming on the development of important interpersonal skills and creativity should be further studied and promoted for the benefits of society.

### Limitations and Ideas for Further Research

The research presented in this paper has some limitations regarding applicability and interpretation. There are several issues we are aware of. First of all, due to the nature of the sample our results should not be considered as representative of the population of the Czech Republic. Second, our research design did not allow us to study the causality of the association.

Third, due to ethical reasons, the Internet questionnaire could not reach respondents below 18 years of age who are likely to be heavy players, as this would have required the informed consent of their parents. Moreover, we suggest that further research may concentrate on the category of players between 15 and 18 years of age to study the association between EC and playing videogames.

Similarly, more work needs to be done on the detailed specification of the amount of time spent playing with respect to gamer groups and the effects of particular types of games on emotional creativity. However, such detailed analysis was out of the scope of this study.

## Figures and Tables

**Table 1:** Number of participants in each gamer group.

Gamer Group	N	%
Group 1. Frequent players—play, on average, more than three hours per day, play daily	45	10.0
Group 2. Regular gamers—play, on average, between one and three hours per day, play daily	58	12.8
Group 3. Infrequent gamers—play, on average, less than one hour per day. Play regularly several times per week.	78	17.2
Group 4. Occasional players. Play less than infrequent players and irregularly.	155	34.2
Group 5. Non-gamers—do not play games	117	25.8

**Table 2:** Descriptive statistics, reliability, and correlations for all continuous and ordinal variables.

	Age	Games	Education	ECI	ECIn	ECIp	ECIae
Age	1.000						
Play time	0.128 **	1.000					
Education	0.424 ***	0.134 **	1.000				
ECI	−0.060	0.105 *	0.013	1.000			
ECIn	−0.157 **	0.007	−0.081	0.823 ***	1.000		
ECIp	0.004	0.193 ***	0.080	0.800 ***	0.511 ***	1.000	
ECIae	0.075	0.080	0.079	0.623 ***	0.174 ***	0.378 ***	1.000
Mean	23.680	3.53	2.170	100.37	46.91	26.08	27.38
Std. deviation	6.356	1.273	0.49312	15.515	8.867	5.601	5.998
Range	18–60	1–5	1–3	51–139	24–68	8–35	10–43
N	453	453	453	453	453	453	453
Cronbach’s alpha (N of Items)				0.85 (30)	0.81 (14)	0.79 (7)	0.74 (9)

Note: Statistical significance for correlations: * *p* < 0.05; ** *p* < 0.01; *** *p* < 0.001 (2-tailed). Education categories: 1- elementary, 2 – secondary, 3-higher. ECI stands for Emotional Creativity Inventory. ECIn, ECIp, ECIae stands for the Novelty, Preparedness, Effectiveness/Authenticity components of ECI.

**Table 3:** The relationship between the emotional creativity inventory (ECI), its subscales novelty (ECIn) and preparedness (ECIp), and the frequency of playing computer games moderated for gender. Results of the multivariate regression analysis.

Dependent Variable	ECI					ECIn					ECIp				
	B		Std. Error	Sig.	VIF	B		Std. Error	Sig.	VIF	B		Std. Error	Sig.	VIF
Constant	97.939	***	4.239	0.000		47.042	***	2.422	0.000		23.486	***	1.516	0.000	
Play time	4.839	**	1.565	0.002	5.36	1.757	*	0.894	0.050	5.36	1.968	***	0.560	0.000	5.36
Play time * Gender	−5.588	**	1.843	0.003	3.93	−3.095	**	1.053	0.003	3.93	−2.095	**	0.659	0.002	3.93
Age	−0.166		0.123	0.179	1.39	−0.190	**	0.070	0.007	1.39	−0.023		0.044	0.607	1.39
Gender	2.134		1.882	0.257	1.81	2.300	*	1.075	0.033	1.81	1.372	*	0.673	0.042	1.81
Secondary education	4.426	*	2.174	0.042	2.29	1.807		1.242	0.146	2.29	1.258		0.778	0.106	2.29
Higher education	4.032		2.851	0.158	2.84	0.334		1.629	0.837	2.84	1.762		1.020	0.085	2.84
Model Sig.	0.000					0.000					0.000				
R^2^	0.060					0.690					0.096				
N	453					453					453				

*** significant at the 0.001 level (2-tailed). ** significant at the 0.01 level (2-tailed). * significant at the 0.05 level (2-tailed). ECI denotes the ECI total score, ECIn and ECIp denote the ECI subscale novelty and preparedness, respectively. Reference category for education: primary education. Play time is transformed taking a smoothing spline approach (Spline Ordinal, Degree 2, Interior Knots 2).

**Table 4:** The association of the novelty component of ECI (ECIn) and play time in women. Results of the multivariate regression analysis.

Dependent Variable	ECIn Women				
	B		Std. Error	Sig.	VIF
Constant	50.419	***	2.137	0.000	
Play time	−1.425	**	0.537	0.008	1.068
Age	−0.189		0.074	0.011	1.313
Secondary education	3.296		1.797	0.068	3.414
Higher education	1.252		2.070	0.546	3.857
Model Sig.	0.005				
R^2^	0.046				
N	301				

*** significant at the 0.001 level (2-tailed). ** significant at the 0.01 level (2-tailed). * significant at the 0.05 level (2-tailed). Play time is transformed taking a smoothing spline approach (Spline Ordinal, Degree 2, Interior Knots 2). The sample of women only.

**Table 5:** The relationship between the effectiveness and authenticity components of the emotional creativity inventory (ECIea, ECIe, ECIa), and the frequency of playing computer games moderated for gender. Results of the multivariate regression analysis.

Dependent Variable	ECIea					ECIa					ECIe				
	B		Std. Error	Sig.	VIF	B		Std. Error	Sig.	VIF	B		Std. Error	Sig.	VIF
Constant	27.411	***	1.671	0.000		11.813	***	0.918	0.000		12.896	***	0.900	0.000	
Play time	1.115		0.617	0.071	5.36	0.102		0.339	0.764	5.36	0.990	**	0.332	0.003	5.36
Play time * Gender	−0.397		0.727	0.585	3.93	0.394		0.399	0.324	3.93	−0.666		0.392	0.090	3.93
Age	0.047		0.049	0.332	1.39	0.049		0.027	0.066	1.39	0.030		0.026	0.252	1.39
Gender	−1.538	*	0.742	0.039	1.81	−0.545		0.407	0.181	1.81	−1.471	***	0.400	0.000	1.81
Secondary Education	1.362		0.857	0.113	2.29	0.435		0.471	0.356	2.29	0.498		0.462	0.281	2.29
Higher Education	1.935		1.124	0.086	2.84	0.314		0.617	0.611	2.84	1.170		0.606	0.054	2.84
Model Sig.	0.012					0.029					0.000				
R2	0.032					0.028					0.500				
N	453					453					453				

*** significant at the 0.001 level (2-tailed). ** significant at the 0.01 level (2-tailed). * significant at the 0.05 level (2-tailed). ECIea denotes the ECI subscale effectiveness/authenticity; ECIe and ECIa denotes the ECI subscales effectiveness and authenticity, respectively. Play time is transformed taking a smoothing spline approach (Spline Ordinal, Degree 2, Interior Knots 2).
